# Microbial Assembly and Stress-Tolerance Mechanisms in Salt-Adapted Plants Along the Shore of a Salt Lake: Implications for Saline–Alkaline Soil Remediation

**DOI:** 10.3390/microorganisms13081942

**Published:** 2025-08-20

**Authors:** Xiaodong Wang, Liu Xu, Xinyu Qi, Jianrong Huang, Mingxian Han, Chuanxu Wang, Xin Li, Hongchen Jiang

**Affiliations:** 1 State Key Laboratory of Geomicrobiology and Environmental Changes, China University of Geosciences, Wuhan 430074, China; shaodong@cug.edu.cn (X.W.); xuliuu@cug.edu.cn (L.X.); hanmingxian@cug.edu.cn (M.H.); 2 School of Environmental Science, China University of Geosciences, Wuhan 430074, China; 20231002846@cug.edu.cn; 3 School of Life Sciences, Henan University, Kaifeng 475001, China; 4 College of Life Sciences, Yuncheng University, Yuncheng 044000, China; wangchuanxu@ycu.edu.cn; 5 Shanxi Key Laboratory of Yuncheng Salt Lake Ecological Protection and Resource Utilization, Yuncheng University, Yuncheng 044000, China; 6 Department of Biology, Xinzhou Normal University, Xinzhou 034000, China; lixin1981@xznu.edu.cn

**Keywords:** salt-adapted plant rhizosphere microbiome, total organic carbon, plant growth-promoting rhizobacteria (PGPR), saline-alkaline soil remediation, salt tolerance mechanisms, Yuncheng salt lake

## Abstract

Investigating the microbial community structure and stress-tolerance mechanisms in the rhizospheres of salt-adapted plants along saline lakes is critical for understanding plant–microbe interactions in extreme environments and developing effective strategies for saline–alkaline soil remediation. This study explored the rhizosphere microbiomes of four salt-adapted species (*Suaeda glauca*, *Artemisia carvifolia*, *Chloris virgata*, and *Limonium bicolor*) from the Yuncheng Salt Lake region in China using high-throughput sequencing. Cultivable salt-tolerant plant growth-promoting rhizobacteria (PGPR) were isolated and characterized to identify functional genes related to stress resistance. Results revealed that plant identity and soil physicochemical properties jointly shaped the microbial community composition, with total organic carbon being a dominant driver explaining 17.6% of the variation. *Cyanobacteria* dominated low-salinity environments, while *Firmicutes* thrived in high-salinity niches. Isolated PGPR strains exhibited tolerance up to 15% salinity and harbored genes associated with heat (*htpX*), osmotic stress (*otsA*), oxidative stress (*katE*), and UV radiation (*uvrA*). Notably, *Peribacillus* and *Isoptericola* strains demonstrated broad functional versatility and robust halotolerance. Our findings highlight that TOC (total organic carbon) plays a pivotal role in microbial assembly under extreme salinity, surpassing host genetic influences. The identified PGPR strains, with their stress-resistance traits and functional gene repertoires, hold significant promise for biotechnological applications in saline–alkaline soil remediation and sustainable agriculture.

## 1. Introduction

Salinization and alkalinization of arid and semi-arid terrains constitute some of the most formidable threats to global food security and ecological resilience. Projections indicate a 50% increase in the global area affected by salinization by 2050, concurrent with a rising global population of 9.7 billion, thereby escalating food demand. The prevalence of yield losses in glycophytic crops under saline conditions necessitates the development of effective remediation strategies [1]. Against this backdrop, enhancing crop salt tolerance has become a critical research priority for achieving sustainable agriculture and ensuring global food security.

Salt-adapted plants, including halophytes and salt-tolerant species, exhibit remarkable physiological and biochemical adaptations enabling survival in hypersaline environments [2]. Although the intrinsic physiology of these plants contributes to their survival, a growing body of evidence supports the vital role of symbiotic relationships with their rhizosphere microbiota in enhancing salt tolerance and fostering successful growth [3,4]. These plant–microbe interactions provide promising biological strategies for restoring degraded saline soils and optimizing agricultural productivity. The rhizosphere, the narrow zone of soil immediately surrounding the plant roots, is shaped by both plant species and soil characteristics [5]. Different plant species selectively enrich specific microbial taxa through root exudates, and this recruitment can exhibit highly specific mechanisms [6]. Among the soil parameters, salinity acts as a major environmental filter, selecting for salt-tolerant microbial lineages and subsequently influencing community structure and diversity [7]. While salinity is often the primary driver, nutrient availability exerts important influences on community composition [8].

Metagenomic analyses of saline soils have consistently identified *Proteobacteria*, *Actinobacteria*, and *Bacteroidota* as the dominant bacterial phyla [9]. However, comprehensive understanding of the microbial ecology in hypersaline systems such as salt lakes remains limited, due to the fact that much research has focused on low-to-moderate salinity conditions, such as coastal wetlands and salt-affected agricultural soils [10,11,12]. Salt lakes, which are characterized by exceeding 20% salinity and a strongly alkaline pH, present a more highly selective environment [13]. Their distinct selective pressures may shape rhizosphere microbial communities to develop unique taxonomic compositions, as well as specialized functional attributes. Salt lake habitats can serve as ideal natural laboratories for investigating microbial adaptation, ecological specialization, and novel stress-resistance mechanisms. Therefore, studies analyzing halophyte microbiomes in highly saline environments remains one of the key areas for investigation.

Microorganisms residing in the rhizosphere are increasingly recognized as pivotal drivers of plant adaptation to abiotic stresses [14]. Of these, PGPR colonize the roots of plants and enhance plant performance under adverse conditions [15]. These beneficial bacteria mitigate salt stress by multiple functional mechanisms, including siderophore production, nitrogen fixation, phytohormone (e.g., indole-3-acetic acid, IAA) biosynthesis, and the secretion of exopolysaccharides [16]. These traits help contribute to the reduction of toxic ions, solubilization of nutrients, and stabilization of soil aggregates, thereby promoting plant growth under saline conditions [17,18,19]. Some PGPR strains have also demonstrated high functional redundancy, simultaneously expressing multiple plant-beneficial traits that act synergistically to alleviate stress and promote growth. In addition, certain PGPR, such as those belonging to the genus *Arthrobacter*, harbor a wide array of genes associated with oxidative stress response, osmotic regulation, and temperature tolerance, enabling survival in harsh conditions [7]. A number of studies have highlighted the considerable potential for using PGPR in sustainable agriculture and soil remediation. For example, inoculation with *Bacillus subtilis* has been shown to increase wheat yield by approximately 18% [20]. Similarly, Mayak et al. [21] demonstrated that PGPR strains isolated from saline riverbeds enhanced the salt tolerance of tomato plants. Despite the growing interest in PGPR, the diversity, functional potential, and stress-mitigation capacity of PGPR under extreme saline–alkaline conditions remain poorly understood, especially in the context of inland salt lake ecosystems. Unraveling these ecological roles will clarify the adaptive strategies developed by PGPR and also their potential applications in enhancing plant tolerance to salt stress.

To address the gaps in knowledge regarding PGPR in highly saline habitats, we propose the following hypotheses: (1) habitat-specific environmental filters sculpt distinct rhizosphere microbiomes of salt-adapted plants, with hypersaline conditions exerting strong selective pressures that reduce alpha diversity; (2) soil nutrients serve as a critical determinant of microbial community composition; (3) PGPR associated with salt-adapted plants possess high redundancy and unique stress-resistance genes that confer enhanced tolerances to salinity, oxidative stress, and nutrient scarcity, making them promising candidates for biotechnological applications.

To test these hypotheses, we integrated high-throughput 16S rRNA gene sequencing, culture-based isolation, and functional gene analyses to characterize the rhizosphere microbiomes of four salt-adapted species—*Suaeda glauca*, *Artemisia carvifolia*, *Chloris virgata*, and *Limonium bicolor*—from the Yuncheng Salt Lake region, China. The objectives of this study are to (1) explore the rhizosphere microbial diversity associated with four salt-adapted plant species; (2) identify the key environmental drivers, including pH, salinity, TOC, TP, and TN, that govern the assembly of rhizosphere microbial communities; and (3) isolate and characterize salt-tolerant PGPR strains possessing multifunctional stress-resistance traits, with an emphasis on identifying potential genes involved in heat resistance, osmoprotection, antioxidation, and UV radiation resistance.

## 2. Materials and Methods

### 2.1. Description of the Study Area

This research was conducted in Yuncheng city, situated in the southwestern region of Shanxi Province in China. The study site was located within the Yuncheng Salt Lake (105°41′–106°58′ E/34°54′–35°04′ N), a national nature reserve known as the “Dead Sea of China”, covering an area of 120 km^2^ (Figure S1a) [22]. The landscape is characterized as a closed terrain with a flat topography and shallow lake features, exhibiting geomorphologic features indicative of salt deposition. The area experiences distinct seasonal climatic conditions. Certain sections within the reserve exhibit extremely high salinity and alkalinity. The climate is classified as a warm-temperate, semi-humid continental monsoon climate, with an average annual temperature of 13.6 °C and an average annual precipitation of 550 mm. Notably, annual evapotranspiration exceeds 2000 mm [23].

### 2.2. Site Selection and Soil Collection

In this study, a comprehensive survey was conducted of all plant life along the littoral zone of the salt lake. Subsequently, the four most prevalent halophytic species within the protected area were identified and selected for further analysis: *Suaeda glauca*, *Artemisia carvifolia*, *Chloris virgata*, and *Limonium bicolor* (Figure S1b). *S. glauca* specimens were categorized into two distinct ecotypes based on their habitat: those inhabiting regions with lower salinity and those found in highly saline environments. For each species, three individual plants exhibiting optimal growth characteristics were randomly selected from their respective areas of maximal abundance. The salinity range of the lacustrine water surrounding the sampled plants ranged from 9.62% to 29.6% [13]. Entire root systems were carefully excavated, preserving their vertical orientation. Rhizospheric soil was then extracted from the root matrix using an oscillatory technique. After carefully removing extraneous materials with forceps, the soil samples were transferred to sterile sampling bags and immediately transported to the laboratory under cryogenic conditions. Upon arrival, each sample was partitioned into three aliquots: one was stored at −80 °C for subsequent high-throughput sequencing; another was air-dried for physicochemical analysis; and the third aliquot was refrigerated at 4 °C for the isolation and purification of rhizosphere microorganisms.

### 2.3. Determination of Rhizosphere Soil Physicochemical Properties

Five distinct sets of soil samples, each consisting of three independent replicates, were subjected to analytical examination to ensure robustness and reproducibility. The suite of soil physicochemical properties analyzed in this study included pH, total organic carbon (TOC), total nitrogen (TN), total phosphorus (TP), and salinity. pH was measured using a pH meter (Ferrymorse-Seed Company, Norton, MA, USA) on suspensions of air-dried soil homogenized with distilled water at a volumetric ratio of 1:2.5 [24]. TP contents were determined using molybdenum–antimony–D-isoascorbic acid colorimetry (MADAC) [25]. Soil salinity was determined by measuring the electrical conductivity (EC) of a soil-to-water suspension (1:5 *w*/*v*) using a conductivity meter [26]. Concurrently, the contents of TOC and TN were determined using a multi TOC/TN^®^ 2100S TOC analyzer (Analytik Jena, Jena, Germany).

### 2.4. DNA Extraction, PCR Amplification, and Illumina MiSeq Sequencing

DNA was extracted from the soil samples using the Fast DNA SPIN Kit (MP Biomedical, Santa Ana, CA, USA), following the manufacturer’s protocol. The quality of the extracted DNA was visually confirmed using 1% agarose gel electrophoresis. The purity and concentration of the DNA were assessed spectrophotometrically using an ultra-micro spectrophotometer (K2800, KAIAO, Beijing, China). The V3–V4 hypervariable region of the bacterial 16S rRNA genes was amplified using polymerase chain reaction (PCR) using the primers 515F (5′-GTGCCAGCMGCCGCGGGTAA-3′) and 806R (5′-GGACTACHVGGGGTWTCTAAT-3′). PCR amplifications were carried out in a 50 μL reaction volume, containing 5 μL of 10× buffer, 4 μL of 2.5 mM dNTP, 0.3 μL of rTaq DNA polymerase, 1 μL of F/R inner primers (10 μM), 1 μL of template DNA, and 37.7 μL of sterile deionized water. PCR products were visualized and gel-extracted following agarose gel electrophoresis, and then purified using the AxyPrep DNA Gel Extraction Kit (CORNING LIFE SCIENCES, Suzhou, China).

The resulting amplicons were submitted to Guangdong Magigene Biomedical Technology Co., Ltd. (Foshan, China) for paired-end sequencing (2 × 250 bp) on an Illumina MiSeq platform. The raw sequencing data have been deposited in the Sequence Read Archive (SRA) database of the National Center for Biotechnology Information (NCBI) under the accession number PRJNA1269453. The raw paired-end sequencing data were analyzed using the UPARSE pipeline [27]. QIIME2 ver. 2023.02 [28] was employed for demultiplexing the sequences. During demultiplexing, reads containing ambiguous bases (N) or failing to match the assigned barcodes and primers were discarded. The remaining reads were processed with DADA2 [29] in QIIME2, including quality filtering, error-correction, and the construction of a feature table representing amplicon sequence variants (ASVs). Reads containing more than three consecutive bases with a Phred quality score below 30 were removed, and only reads where at least 75% of the bases maintained a Phred score of 30 or greater were retained [30]. Chimeric sequences were identified and removed using uchime-denovo in the USEARCH program [31]. In the resulting feature table, ASVs replaced operational taxonomic units (OTUs) as the marker gene reporting units [32]. Singletons (ASVs represented by only a single sequence across the entire dataset) were removed to minimize the impact sequencing artifacts. Taxonomic classification of the representative ASV sequences was performed using the RDP Classifier algorithm in QIIME2, employing the SILVA 138 database with a minimum confidence threshold of 80% [33]. ASVs classified as chloroplast sequences were excluded prior to downstream analysis. Ultimately, the obtained ASV table was used for subsequent analyses of microbial community diversity.

### 2.5. Isolation and Characterization of Rhizosphere Soil Bacteria

To isolate bacteria from the rhizosphere, five grams of fresh rhizosphere soil were aseptically added to a conical flask containing 45 mL of sterile saline water. This mixture was vigorously shaken at 150 rpm for 2 h to create a 10-fold dilution of the soil suspension. Serial dilutions were then carried out, generating a range of soil suspensions from 10^−2^ to 10^−5^. Aliquots of 100 μL from the 10^−4^ and 10^−5^ dilutions were inoculated onto nine different agar culture media (Table S1). After incubation at 28 °C for 7 days, bacterial colonies exhibiting distinct morphologies (based on color, size, shape, and colony morphology) were selected and transferred to fresh LB agar plates for further purification.

The purified bacterial isolates were identified through 16S rRNA gene sequencing. DNA was extracted using the Chelex 100 method. Purity and concentration assessment, PCR amplification, and PCR product purification followed the procedures outlined in the previous section. The purified PCR products were then submitted to Sangon Co., Ltd. (Shanghai, China) for Sanger sequencing. The resulting sequences were compared against the EzBioCloud Database [34] for identification of the bacterial isolates. All purified strains were preserved as glycerol stocks (20% *v*/*v* glycerol) at −80 °C for long-term storage.

### 2.6. Screening of Plant Growth Promoting Bacteria

Based on the phylogenetic relationships of the closest related taxa, a subset of 35 representative strains was selected from the 121 isolates to assess halotolerance and screen for plant growth-promoting (PGP) capabilities. These strains were inoculated into R2A liquid medium supplemented incrementally with sodium chloride (2%, 3.5%, 5%, 7%, and 15% *w*/*v*) and cultivated for seven days. Growth kinetics were evaluated spectrophotometrically by measuring the optical density at 600 nm (OD_600_).

To identify strains exhibiting PGP traits, six assays were implemented. These included the quantification of indole 3-acetic acid (IAA) production, phosphate solubilization, carboxymethyl cellulose (CMCase) production, siderophore biosynthesis, nitrogen fixation, and exopolysaccharide (EPS) elaboration. IAA production was quantified using a colorimetric assay, as modified from a previously established protocol [35]. CMCase activity was assessed using a plate assay involving Congo red staining [36] with minor adaptations. Briefly, plates were immersed in a 0.5% (*w*/*v*) Congo red solution for 60 min followed by destaining with a 1 mol/L NaCl solution for 30 min. Nitrogen fixation and phosphate solubilization capabilities were determined according to the methodology described in reference [37]. Specifically, strains were spot inoculated onto NBRIP medium (for inorganic phosphate) and Mongina medium (for organic phosphate) and incubated at 30 °C for 72 h. Strains showing a clear halo around the colony were considered to have phosphorus-solubilizing activity. EPS production was evaluated according to the previously described method detailed previously [36].

### 2.7. Data Processing

Statistical analyses were performed utilizing IBM SPSS Statistics ver. 29.0.1.0 and R ver. 4.3.2. Prior to conducting α-diversity and β-diversity analyses, the feature table was rarefied to an equal sequencing depth (*n* = 145,196 reads per sample), with this resampling process repeated 1000 times to ensure robustness. The “vegan” package was employed to compute the measures of ASV richness, as well as alpha diversity indices, including Shannon, Simpson, and equitability. Taxonomic composition and relative abundance profiles for each sample were examined using R. One-way analysis of variance (ANOVA), followed by Tukey’s honest significant difference (HSD) post hoc test, was conducted using IBM SPSS to identify statistically significant differences in soil chemical properties and alpha diversity metrics.

To elucidate the structural composition of the microbial communities, principal coordinate analysis (PCoA) based on Bray–Curtis dissimilarities was performed utilizing the “vegan” package [38]. Redundancy analysis (RDA) and heatmaps displaying Spearman correlation coefficients were generated to elucidate associations between rhizosphere soil characteristics and microbial community structure [39]. Differential abundance of the taxa among sample groups was assessed using LEfSe (Linear Discriminant Analysis Effect Size), with thresholds set at an LDA score > 4 and a significance level *p* < 0.05 [40]. Genomic sequences, downloaded from the NCBI database based on the closest phylogenetic matches, were aligned against the Kyoto Encyclopedia of Genes and Genomes (KEGG) database using MMseqs v0.0.1 [41], with an E-value cutoff of <1 × 10^−15^, to construct a gene abundance profile. Phylogenetic relationships were inferred from the 16S rRNA sequences via MEGA11 [42], and the resulting phylogenetic trees were visualized and annotated using Itol (v.6) [43]. All graphical representations, including box plots, upset plots, histograms, and heatmaps, were generated utilizing R to facilitate comprehensive data visualizations and interpretation.

## 3. Results

### 3.1. Rhizosphere Soil Properties

The physicochemical properties of the five rhizosphere soil samples varied significantly (Table 1). All samples were weakly alkaline, with pH values ranging from 8.12 to 8.56. SGR exhibited the highest EC, while SGG showed the lowest (*p* < 0.05). CVA had the highest TOC, TP, and TN concentrations; TOC was significantly higher in CVA than in the other four samples. LBR and SGG had the lowest TOC and TP contents, respectively, and SGR had the lowest TN content. TP content did not significantly differ between samples, except for CVA. Notably, the two *Suaeda glauca* samples (SGG and SGR) differed significantly in all indices except TOC and TP.

### 3.2. Diversity of Rhizosphere Soil Microbial Communities

**Figure 1:**
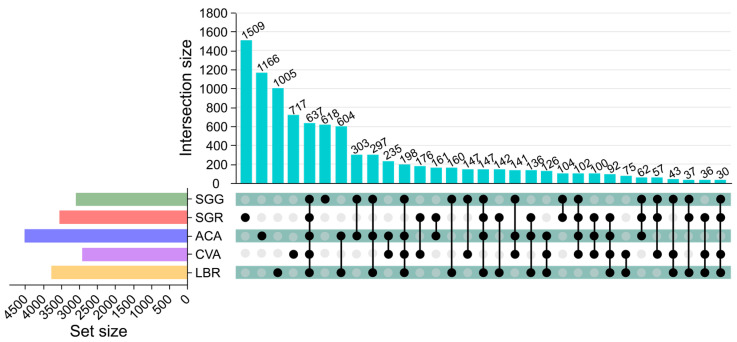
Upset diagram illustrating the soil bacterial operational taxonomic units (ASVs) in the rhizosphere soil of five plants. The upper vertical bars represent the number of ASVs shared among different plant rhizospheres, with connected black circles below indicating the specific sets included in each intersection and gray circles showing the sets not involved. The horizontal bars display the size of each ASV set.

A total of 9363 ASVs were annotated across all rhizosphere soil samples, with an average of 1873 ASVs per sample. Specifically, SGG, SGR, ACA, CVA, and LBR contained 3183, 3528, 4507, 2912, and 3766 ASVs, respectively. Each soil sample possessed a distinct set of unique ASVs, with SGG, SGR, ACA, CVA, and LBR containing 618, 1509, 1166, 717, and 1005 unique ASVs, respectively. Across all samples, 637 ASVs were common, representing 6.80% of the total ASVs. Notably, SGR had a significantly higher number of unique ASVs compared to the other samples, especially in comparison to SGG (Figure 1).

**Figure 2:**
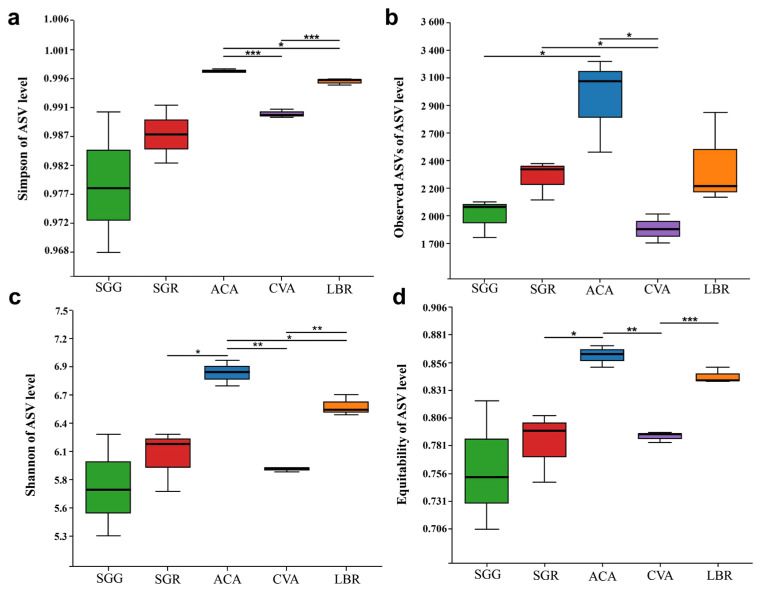
Box plots illustrating the alpha (α) diversity of microbial communities in the rhizosphere soils of five plant species. (**a**) Simpson index; (**b**) the number of observed ASVs; (**c**) Shannon index; (**d**) equitability index. Significant differences between the pairs of plant species are indicated by asterisks (*: *p* < 0.05, **: *p* < 0.01, ***: *p* < 0.001).

Regarding α diversity indices, including the Simpson index, observed ASVs, Shannon index, and equitability, ACA exhibited the highest diversity values, followed by LBR. Conversely, SGG exhibited the lowest values for the Shannon, Simpson, and equitability indices, while its observed ASVs indicated slightly higher richness than SGG. Overall, the α diversity indices of SGR were consistently somewhat higher than those of SGG. Boxplot analyses revealed no statistically significant differences between SGG and SGR across these four diversity indices. However, significant differences in community diversity, as assessed by the Simpson and Shannon indices, were observed among ACA, CVA, and LBR (Figure 2).

### 3.3. Composition of Rhizosphere Soil Microbial Communities

At the phylum level, a total of 44 bacterial phyla were identified in the rhizosphere soils. The dominant phyla, each with relative abundances greater than 1%, included *Proteobacteria*, *Actinobacteriota*, *Bacteroidota*, *Gemmatimonadota*, *Planctomycetota*, *Chloroflexi*, *Acidobacteriota*, and *Myxococcota* (Figure S2a). The composition of microbial taxa varied significantly among the soils associated with different plant species. Notably, even between the two samples (SGG and SGR) from the same plant species (*Suaeda glauca*), there were substantial differences in the relative abundances of *Proteobacteria*, *Chloroflexi*, and *Acidobacteriota*.

At the class level, 108 classes were identified across all samples. Eight classes had relative abundances exceeding 1% in all soil samples: *Gammaproteobacteria*, *Alphaproteobacteria*, *Bacteroidia*, *Actinobacteria*, *Planctomycetes*, *Acidimicrobiia*, *Gemmatimonadetes*, and *Longimicrobia* (Figure S2b). Significant variations in microbial taxa were observed between different soil samples. Specifically, SGR, derived from the same plant species as SGG, showed notably lower abundances of *Gammaproteobacteria* (4.94%), *Rhodothermia* (0.14%), and BSGR-11_terrestrial_group (0.44%). Conversely, SGG displayed significantly higher relative abundances of *Cyanobacteria* (35.36%), *Gemmatimonadetes* (2.05%), *Vicinamibacteria* (1.44%), and *Thermoleophilia* (1.17%).

### 3.4. Structure of Rhizosphere Soil Microbial Communities

**Figure 3:**
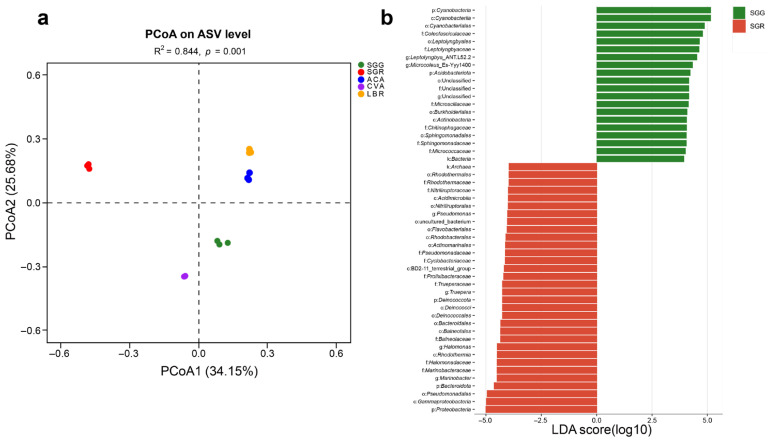
Comparative analysis of the rhizosphere microbial assemblages and taxa exhibiting differential abundance. (**a**) PCoA ordination plot illustrating differences in the species-level microbial community structure across five rhizosphere soil samples. (**b**) LEfSe analysis identifying differentially abundant microbial taxa in the rhizosphere soils of plants grown under the conditions of SGG versus SGR. Taxa with significantly different relative abundances between the two groups were identified using an LDA > 4.

A PCoA based on the Bray–Curtis distance algorithm was performed at the ASV level to assess the microbial community structures across all soil samples. The analysis revealed that the first two principal coordinates (PCoA1 and PCoA2) accounted for 34.15% and 25.68% of the total variation, respectively. The R^2^ value of 0.844 indicated that differences between samples from different groups were substantially greater than the variation within the same group, demonstrating clear separation among sample groups. The PCoA plot showed strong within-group reproducibility and distinct between-group differences for the five soil samples (Figure 3a).

LEfSe analysis with an LDA score threshold greater than four showed differentially abundant microbial taxa in the rhizosphere soils of different plants, identifying potential microbial biomarkers across all rhizosphere samples. Comparison between SGG and SGR revealed that the dominant phyla in SGG were *Cyanobacteria* and *Acidobacteriota*, whereas in SGR, *Proteobacteria*, *Bacteroidota*, and *Deinococcota* were predominant (Figure 3b). Across all samples (SGG, SGR, ACA, CVA, and LBR), a total of 110 taxa showed significant differences (*p* < 0.05), with 19, 26, 19, 33, and 13 taxa unique to each group, respectively. At the phylum level, distinct enrichment patterns were observed. *Cyanobacteria* were notably enriched in SGG (Figure S3). SGR showed a significant enrichment of *Bacteroidota* and *Firmicutes*. In ACA, *Gemmatimonadota*, *Chloroflexi,* and *Planctomycetota* were preferentially enriched, while CVA displayed a significant enrichment of *Actinobacteria*. Meanwhile, *Proteobacteria* dominated the microbial communities in LBR. These results demonstrated clear compositional differentiation among the microbial communities associated with different plant hosts and soil types.

### 3.5. Correlations Between Rhizosphere Soil Physicochemical Properties and Microbial Communities

**Figure 4:**
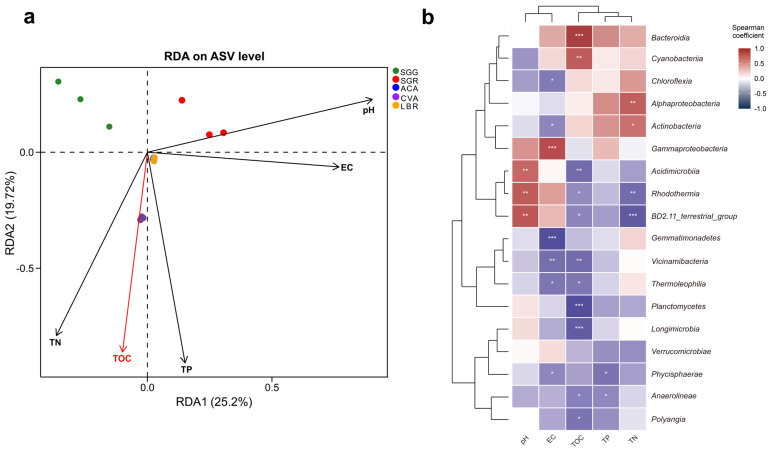
Correlations between soil physicochemical properties and rhizosphere microbial communities. (**a**) RDA illustrating the relationships between soil physicochemical properties and microbial community composition. The red arrows indicate physicochemical factors that significantly influence microbial variation, with their length proportional to the strength of the correlation. (**b**) Spearman correlation analysis examining the relationships between soil physicochemical properties and microbial communities at the class level. Asterisks (*) above the line indicate a statistically significant difference between the compared samples, with * indicating *p* < 0.05, ** indicating *p* < 0.01, and *** indicating *p* < 0.001.

RDA analysis was employed to explore the relationships between environmental variables and rhizosphere microbial community composition (Figure 4a). The first two RDA axes, RDA1 and RDA2, accounted for 25.20% and 19.72% of the total variation, respectively, indicating a substantial influence of measured environmental factors. RDA1 was positively correlated with pH, EC, and TP, suggesting these factors were primary drivers of microbial community structure variation along this axis. RDA2 showed a positive correlation with pH, but a negative correlation with TOC, TP, and TN, further distinguishing the influence of these variables. Additionally, the proximity of ACA and LBR samples in the RDA ordination suggested similar microbial community structures within their rhizospheres. Conversely, the distinct separation of CVA, SGG, and SGR underscored the species-specific differences in their microbial community structures, potentially reflecting variations in root exudate profiles or other plant-mediated factors.

Spearman correlation analysis revealed diverse responses of the microbial community composition (specifically the top 18 class) to environmental factors (Figure 4b, Table S3). Notably, *Bacteroidia* and *Cyanobacteria* exhibited significant and positive associations with TOC (*p* < 0.001), whereas *Planctomycetes*, *Longimicrobia,* and *Polyangia* exhibited significant negative correlations with TOC (*p* < 0.05). *Rhodothermia* and BD2-11_terrestrial_group showed marked positive correlations with pH (*p* < 0.01), yet were inversely related to TOC and TN (*p* < 0.05). *Gammaproteobacteria* displayed a robust positive correlation with EC (*p* < 0.001), while *Chloroflexia*, *Gemmatimonadetes*, *Vicinamibacteria*, *Thermoleophilia,* and *Phycisphaerae* were all significantly positively correlated with EC (*p* < 0.05). *Acidimicrobiia* exhibited a notable positive correlation with pH (*p* < 0.01) and a concomitant negative relationship with TOC (*p* < 0.01). Both *Acidimicrobiia* and *Rhodothermia* were significantly positively correlated with pH (*p* < 0.01), yet negatively correlated with TOC and TN (*p* < 0.05). Additionally, *Phycisphaerae* and *Anaerolineae* showed significant positive correlation with TP (*p* < 0.05). Furthermore, *Alphaproteobacteria* and *Actinobacteria* were markedly positively correlated with TN (*p* < 0.05). Among the measured environmental variables, TOC exhibited the most substantial statistical association with microbial community structure (R^2^ = 0.176, *p* < 0.05, Figure 4a), and showed significant positive or negative correlations with multiple microbial taxa at the class level (Figure 4b). The coefficients of determination (R^2^) and corresponding *p* values for the remaining variables are detailed in Table S2.

### 3.6. Diversity of Culturable Rhizosphere Soil Microorganisms

**Figure 5:**
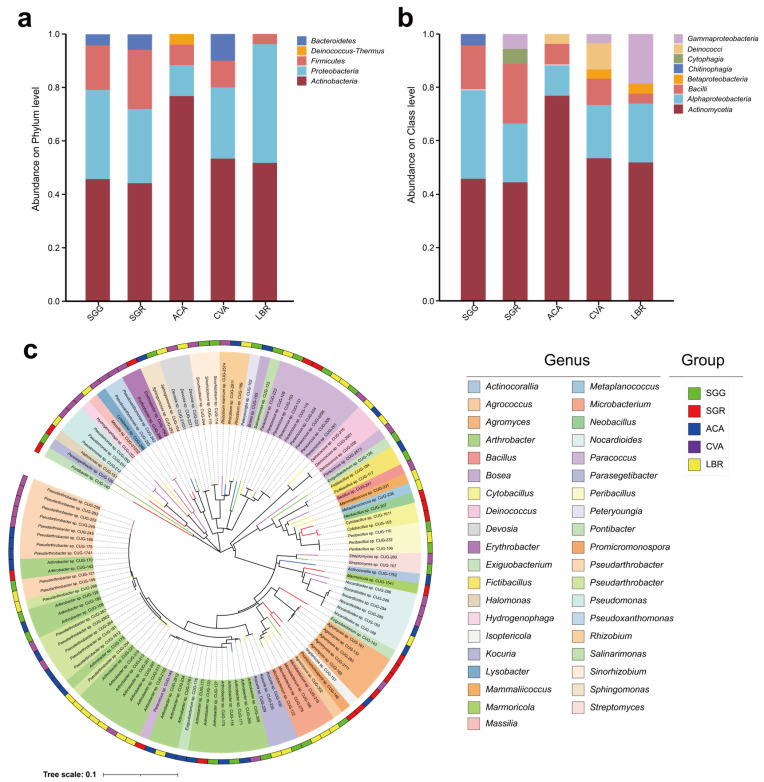
Taxonomic profiling and phylogenetic diversity of the culturable rhizosphere bacteria. Panels (**a**,**b**) represent the relative abundances of culturable microorganisms at the phylum and class levels, respectively, found in the rhizosphere soils of the five investigated plant species. Panel (**c**) represents the phylogenetic tree illustrating 121 bacterial strains isolated from the rhizosphere soils of five plant species. The inner ring, segmented into 39 distinct colors, represents different genera, while the outer ring, colored in five different hues, corresponds to the rhizosphere soil samples. The colors of the outermost branches of the phylogenetic tree match those of the corresponding rhizosphere soil samples.

A total of 121 microbial strains were isolated from all soil samples, spanning five phyla, eight classes, eighteen orders, twenty-six families, thirty-nine genera, and sixty-six species. The five phyla identified were *Actinobacteria*, *Proteobacteria*, *Firmicutes*, *Deinococcus-Thermus*, and *Bacteroidetes* (Figure 5a).

At the class level, *Actinomycetia* and *Alphaproteobacteria* emerged as the predominant taxa across all five soil samples. The dominant classes in the ACA sample were *Actinomycetia* (76.9%), *Alphaproteobacteria* (11.5%), *Bacilli* (7.7%), and *Deinococci* (3.8%) (Figure 5b). In the CVA sample, the prevailing classes included *Actinomycetia* (53.3%), *Alphaproteobacteria* (20%), *Bacilli* (10%), *Deinococci* (10%), *Betaproteobacteria* (3.3%), and *Gammaproteobacteria* (3.3%). For LBR, the main classes comprised *Actinomycetia* (51.9%), *Alphaproteobacteria* (22.2%), and *Gammaproteobacteria* (18.5%). In the SGG sample, the dominant classes were *Actinomycetia* (45.8%), *Alphaproteobacteria* (33.3%), *Bacilli* (16.7%), and *Chitinophagia* (4.2%). Meanwhile, in SGR, the principal classes were *Actinomycetia* (44.4%), *Alphaproteobacteria* (22.2%), *Bacilli* (22.2%), *Cytophagia* (5.6%), and *Gammaproteobacteria* (5.6%) (Figure 5b). In contrast, *Chitinophagia* and *Cytophagia* displayed unique associations with the SGG and SGR samples, respectively (Figure 5b). The genus-level composition of both the rhizosphere microbial communities and the culturable microorganisms was incorporated in Figure S4. The dominant genera identified in the rhizosphere soil samples included *Sphingomonas* (3.4%), *Pseudomonas* (2.3%), *Pontibacter* (1.7%), *Marinobacter* (1.4%), and *Arthrobacter* (1.2%). For the culturable microorganisms, the dominant genera included *Arthrobacter* (19.5%), *Pseudarthrobacter* (12.6%), *Paracoccus* (9.3%), *Agromyces* (5.6%), and *Nocardioides* (4.4%). Figure 5c illustrates the distribution of the 121 strains at the genus level, illustrating their specific associations with each respective soil sample.

### 3.7. Results of Stress Resistance Screening

Among the isolated strains, 35 representatives displayed significant variation in their salt tolerance capabilities, demonstrating overall strong resilience to salinity stress. Specifically, ten strains tolerated salinity levels up to 7%, three strains tolerated 10% salinity, and four strains showed tolerance to 15% salinity (Figure S5).

**Figure 6:**
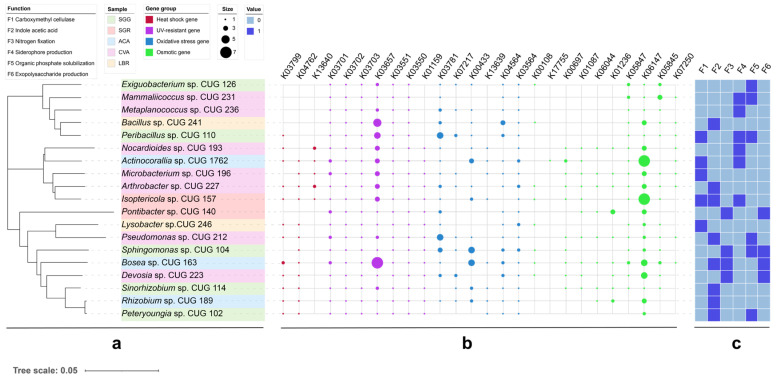
Phylogenetic analysis and stress resistance profiles of PGPR strains. (**a**) Phylogenetic tree of 19 PGPR strains. (**b**) Dot plot showing the presence and distribution of stress resistance genes within each PGPR strain. Dot size may represent gene copy number or relative abundance. (**c**) Heatmap displaying the functional profiles of the PGPR strains related to stress resistance. The color intensity indicates the relative abundance or expression level of the different stress resistance functions.

A total of 19 strains exhibiting plant growth-promoting functions were isolated from all soil samples (Figure 6). Notably, 52.6% of these strains exhibited functional redundancy by possessing multiple growth-promoting traits. Among these, five strains produced cellulase, six demonstrated organic phosphorus solubilization, and eight could synthesize IAA. Further metabolic characterization revealed that cellulase activity was present in five strains, IAA biosynthesis in eight strains, nitrogen fixation ability in four strains, siderophore production in six strains, organic phosphorus solubilization in six strains, and extracellular polysaccharide production in four strains. Representative plate photographs exhibiting nitrogen fixation, siderophore production, phosphate solubilization, and cellulase synthesis capabilities are provided in Figure S6. Genome analysis revealed the presence of multiple stress response-related genes. These include heat shock genes (e.g., *htpX*, *hslR*), UV resistance genes (e.g., *ruvB*, *uvrA-D*), antioxidant genes (e.g., *katE*, *soxR*), and osmoprotectant synthesis genes (e.g., *otsA/B*, *treY/Z*). Detailed KEGG orthology annotations for these genes are provided in Table S4.

## 4. Discussion

### 4.1. Habitat-Specific Selection Drives Rhizosphere Microbial Assembly in Salt-Adapted Plants

Our initial hypothesis posited that the hypersaline environments of the Yuncheng Salt Lake region would sculpt distinct rhizosphere microbiomes in salt-adapted plants and result in a reduced alpha diversity. Our findings supported only the first part of this hypothesis. The predominant bacterial phyla consistently identified across the five rhizosphere soil samples were *Proteobacteria* (32.4%), *Actinobacteria* (13.5%), and *Bacteroidota* (12.5%). These taxa are frequently documented in investigations of saline soil microbiomes and are regarded as fundamental constituents of the microbial assemblages in Chinese saline ecosystems. We hypothesize that diverse salt-adapted plants occupying coastal, saline–alkaline, and desert habitats may selectively recruit core microbial taxa with conserved community compositions, despite potential variability in their diversity and relative abundance contingent upon the specific host plant species.

Analysis of alpha diversity metrics—namely Shannon, Simpson, and equitability indices—exhibited significant variability across rhizosphere soil samples from different plants (Figure 2), with SGR (hyper-saline soil) demonstrating elevated diversity relative to SGG (low-salinity soil). This observation contradicts the expectation that extreme salinity would suppress microbial diversity; however, it is consistent with previous studies demonstrating that salt-adapted plants can selectively recruit specialized microbial consortia [3,4], thereby promoting the enrichment and proliferation of salt-adapted microbial taxa. For instance, SGR harbored 1509 unique amplicon sequence variants (ASVs; Figure 1), suggesting that heightened salinity conditions may selectively favor distinct microbial taxa rather than merely reducing overall diversity.

Furthermore, beta-diversity analyses conducted through principal coordinate analysis (PCoA) demonstrated distinct separation between the rhizospheres of SGG and LBR (Figure 3a), indicating that plant species identity significantly shapes microbial community composition. This observation aligns with the earlier findings [5] that emphasized the critical role of root exudates in structuring rhizosphere microbiomes. However, the notable divergence observed between SGR and SGG—which are the same plant species exposed to different salinity levels—highlights the dominant influence of edaphic (soil-related) factors over the host genetic background in determining microbial assemblages under extreme salinity conditions. LEfSe analysis identified *Cyanobacteria* as biomarkers characteristic of low-salinity *S. glauca* (SGG), whereas members of *Bacteroidetes* and *Firmicutes* were markedly enriched in high-salinity *S. glauca* (SGR) (Figure 3b). These patterns support previous findings that *Cyanobacteria* dominate low-salinity environments due to their capabilities in auxin biosynthesis, siderophore-mediated iron mobilization, and photosynthetic nitrogen fixation, whereas halotolerant taxa such as *Firmicutes* (e.g., *Halanaerobium*) thrive under high-salinity conditions [8,13,44]. We speculate that the significant enrichment of *Bacteroidota* and *Firmicutes* in SGR may play a role in its significantly higher salt tolerance compared to SGG, potentially through mechanisms related to osmoregulation and stress mitigation.

### 4.2. Relationships Between Soil Factors and Rhizosphere Soil Microbial Communities

The results of this study revealed significant differences in the physicochemical characteristics of the rhizosphere soils across the five plant species (Table 1). We propose that these differences may be related to the components of the rhizosphere sediments released by the plants which can contribute to the formation of unique soil microenvironments [7]. Redundancy analysis (RDA) indicated that soil nutrients (e.g., TOC and TN) were strongly correlated with microbial community composition (Table S3). In particular, TOC emerges as a pivotal factor shaping microbial community assembly within salt lake rhizospheres. TOC accounted for the largest proportion of variance (17.6%) in microbial community composition (Figure 4a), aligning with studies in desert soils where recalcitrant organic carbon influences microbial network structure [24]. Our result indicates that in the rhizosphere of salt-adapted plants, organic carbon content may exert a stronger influence on microbial community composition than pH and EC. A significant portion of the microbial phylum and TOC content were negatively correlated. We speculate that may be due to the fact that organic carbon in the studied region predominantly exists in recalcitrant forms, such as mineral-bound states or difficult-to-degrade humus, which are not readily bioavailable to microorganisms [17,45]. Notably, *Cyanobacteria* exhibited a strong positive correlation with TOC (ρ = 0.71, *p* < 0.001), consistent with their known role in degrading complex organic substrates (Figure 4b) [46,47]. Moreover, some taxa may be better adapted to oligotrophic conditions and thus become less competitive in environments with high TOC.

In contrast, members of *Acidimicrobiia* showed a negative correlation with TOC (ρ = −0.68, *p* < 0.05), implying niche differentiation driven by varying carbon availability. While *Acidimicrobiia* are predominantly associated with acidic soils [48], their prevalence in circumneutral salt lake soils suggests metabolic versatility. This observation challenges the conventional view that *Acidimicrobiia* are strictly acidophilic and underscores the importance of functional studies to elucidate the ecological roles of understudied taxa in saline environments. Taken together, our findings support the second hypothesis that soil nutrients, especially TOC, serve as a critical determinant of microbial community composition.

### 4.3. Functional Traits of Culturable PGPR and Stress Resistance Mechanisms

The third hypothesis postulated that plant growth-promoting rhizobacteria (PGPR) associated with salt-adapted plants possess high functional redundancy and distinct stress-resistance genes. Screening of 121 isolates identified 19 strains exhibiting plant growth-promoting (PGP) traits, including siderophore production, exopolysaccharide (EPS) secretion, and indole-3-acetic acid (IAA) biosynthesis (Figure 6). Notably, strains affiliated with *Halomonas* and *Mammaliicoccus* demonstrated tolerances of up to 15% salinity, exceeding the tolerance thresholds documented for coastal salt-adapted plants [10,11,49]. These findings substantiate the hypothesis that salt lake environments select for extremophilic PGPR. Further investigation of the culturable microbial community revealed a disparity between the phylogenetic composition of the rhizosphere and cultured isolates. While cultivation recovered numerous microbial taxa, only a limited number of dominant genera, including *Sphingomonas*, *Pseudomonas*, and *Arthrobacter*, were successfully cultured (Figure S4). This discrepancy suggests that the cultivation methods used in this study may have selectively enriched for a subset of the full rhizospheric diversity. Furthermore, three bacterial isolates recovered during this study could potentially represent novel species: *Pontibacter beigongshangensis* [50], *Salinarimonas ramus* [51], and *Marmoricola terrae* [52]. However, only *Pontibacter* sp. CUG 140 exhibited plant growth-promoting traits.

Genomic analysis based on KEGG gene annotation revealed the potential stress-resistance genes, including *htpX* (encoding a heat shock protein), *uvrA-D* (involved in UV repair), and *otsA/B* (responsible for trehalose synthesis) (Table S4). Compared to PGPR strains isolated from low-salinity rhizosphere soils, those obtained from high-salinity environments (under high osmotic stress) exhibited an upregulation of otsA/B (Figure 6), a pattern consistent with the findings of Etesami and Beattie [53], who established a link between trehalose accumulation and osmotic balance in salt-tolerant rhizobia. However, the absence of *betA* (encoding choline dehydrogenase) in most isolates implicates alternative osmoadaptation strategies, potentially involving glycine betaine synthesis via the *codA* pathway (which was not investigated in this study).

### 4.4. Implications of Rhizosphere Microorganisms in Salt Lake Ecosystems and Agricultural Applications

The distinct microbial assemblages identified in this study (e.g., *Cyanobacteria*-dominated SGG versus *Firmicutes*-enriched SGR) suggest niche specialization driven by varying salinity levels. Such compartmentalization may contribute to the stability of ecosystem functioning by ensuring metabolic complementarity under fluctuating environmental conditions [14]. For instance, *Cyanobacteria* thriving in low-salinity zones could enhance nitrogen fixation, while *Firmicutes* inhabiting high-salinity zones could contribute to the decomposition of organic matter.

From an applied perspective, the PGPR strains characterized in this work (e.g., *Peribacillus* sp. CUG 110 and *Isoptericola* sp. CUG 157) offer potential for bioaugmentation in saline soils. Their multifunctional PGP traits (e.g., phosphate solubilization and EPS production) align with perspectives proposed by Bodur et al. [54] for maintaining ecosystem stability. However, the variable expression of stress-resistance genes (such as *katE*, encoding catalase) under field conditions merits further investigation to ensure their effectiveness in real-world applications. These results support our third hypothesis that these strains exhibit the high functional redundancy and presence of stress-resistance genes, making them promising candidates for plant growth-promoting inoculants.

### 4.5. Limitations and Future Directions

Although this study significantly advances our understanding of rhizosphere microbiomes in salt-adapted plants, several limitations warrant acknowledgment. Firstly, the relatively small sample size (*n* = 5) restricts the broader generalizability of our findings. Future research should address this by incorporating spatially explicit sampling across broader hydrological gradients within the Yuncheng Salt Lake. Secondly, the RDA explained only about 44.92% of the variation (RDA1 + RDA2), indicating that unmeasured environmental parameters, such as specific soil physicochemical properties not captured in this study, likely contribute substantially to shaping the microbial community. A more comprehensive characterization of soil factors is therefore essential for a complete understanding of these complex interactions. Thirdly, the inference of stress-resistance genes relied on genome annotations from closely related NCBI strains. Direct experimental validation, ideally through transcriptomic and proteomic analyses, is crucial to confirm the actual expression and functional activity of these genes under saline stress conditions. Such an approach would provide robust evidence for their roles in microbial adaptation. Finally, the precise mechanisms by which the identified stress-resistant and growth-promoting rhizobacteria exert their beneficial effects on plants remain to be fully elucidated. Future investigations should integrate metabolomics with metagenomics to unravel the intricate plant–microbe signaling pathways in these saline environments. The PGPR strains identified in this study, selected for their demonstrated salt tolerance, inferred stress-resistance genes, and functional redundancy, represent promising candidate inoculants. Their efficacy in promoting crop growth under saline–alkaline conditions will be rigorously evaluated in subsequent experiments, accompanied by transcriptomic analyses of the inoculated crops to definitively clarify the underlying growth-promoting mechanisms.

## 5. Conclusions

This study illuminates the assembly rules and functional characteristics of rhizosphere microbiomes associated with salt-adapted plants in China’s Yuncheng Salt Lake region. *Suaeda glauca* and *Limonium bicolor* hosted divergent rhizosphere microbiota, with *Cyanobacteria* dominating low-salinity niches and *Firmicutes* thriving in high-salinity zones, reflecting niche differentiation driven by salinity gradients and root exudate profiles. Notably, TOC explained 17.6% of the microbial compositional variation, highlighting its pivotal role in stabilizing ecosystem functions through metabolic complementarity. This finding suggests that, once salinity reaches a certain threshold, soil nutrient availability becomes a key environmental driver. Culturable PGPR isolated from salt-adapted plants rhizospheres exhibited high functional redundancy, exceptional stress tolerance (e.g., 15% salinity), and encoded multifunctional genes for heat resistance, osmoprotection, antioxidation, and UV radiation resistance, suggesting biotechnological potential for saline soil remediation. In summary, this dual approach research study bridges critical knowledge gaps in understanding plant–microbe co-adaptation in extreme environments and offers practical insights for harnessing microbiomes of salt-adapted plants to improve agricultural sustainability in saline–alkaline regions.

## Figures and Tables

**Table 1:** Key physicochemical properties of the rhizosphere soils in this study.

Samples	pH	EC (mS/cm)	TOC (mg/kg)	TP (mg/kg)	TN (mg/kg)
SGG	8.12 ± 0.01 ^d^	6.48 ± 0.16 ^d^	358.70 ± 15.02 ^b^	46.13 ± 0.46 ^b^	247.38 ± 20.41 ^bc^
SGR	8.56 ± 0.03 ^a^	21.12 ± 0.20 ^a^	350.28 ± 3.53 ^b^	52.53 ± 2.31 ^b^	221.19 ± 6.44 ^a^
ACA	8.29 ± 0.02 ^b^	5.12 ± 0.20 ^e^	195.91 ± 5.63 ^c^	49.87 ± 15.79 ^b^	233.57 ± 15.57 ^ac^
CVA	8.20 ± 0.05 ^c^	13.04 ± 0.17 ^c^	1550.00 ± 6.28 ^a^	76.67 ± 3.70 ^a^	285.00 ± 4.69 ^a^
LBR	8.16 ± 0.03 ^cd^	17.12 ± 0.16 ^b^	192.50 ± 12.35 ^c^	58.13 ± 5.33 ^b^	258.81 ± 11.89 ^b^

Statistically significant differences (*p* < 0.05) among plant soil types are indicated by different lowercase letters.

## Data Availability

The original contributions presented in this study are included in the article/Supplementary Materials. Further inquiries can be directed to the corresponding authors.

## Supplementary Materials

The following supporting information can be downloaded at: https://www.mdpi.com/article/10.3390/microorganisms13081942/s1, Figure S1: Information about collected samples. (a) Spatial distribution of the sampling sites. The location of each rhizosphere soil sample is denoted by a distinct color-coded marker. (b) Photographic representations of the host plants associated with the five collected rhizosphere soil samples in the Yuncheng Salt Lake region. Abbreviations above each image correspond to the respective plant rhizosphere soil samples; Figure S2: Relative abundances of the major bacteria phyla (a) and class (b) identified in the rhizosphere soils of five plant species; Figure S3: LEfSe analysis of the rhizosphere soils among five plants. Different colored regions represented different constituents. The inner to outer circle corresponds to the level of the phylum to the genus. (Significant differences were determined at LDA > 4); Figure S4: Taxonomic composition of rhizosphere microbial communities at the genus level. (a) Relative abundance of bacterial genera in rhizosphere soils associated with five plant species. (b) Relative abundance of culturable microbial isolates obtained from five rhizosphere soil samples; Figure S5: The OD600 values of 35 representative strains were measured after seven days of cultivation under five salinity gradients (2%, 3.5%, 5%, 7%, 10%, and 15%); Figure S6: Representative plate photographs of bacterial strains exhibiting (a) siderophore production, (b) nitrogen fixation, (c) phosphate solubilization, and (d) cellulase synthesis capabilities; Table S1: Formulations of nine culture media for microbial enrichment in rhizosphere soil; Table S2: Variation partitioning was used to assess the relative contribution of soil salinity, pH, and moisture to microbial community variation. The values of RDA1 and RDA2 correspond to the cosine of the angle between the environmental variable arrows and the ordination axes. r^2^ measures the extent to which each environmental factor contributes to the variation in species distribution, while *P* (*p*-value) denotes the significance level of the correlation; Table S3: The Spearman correlation value between soil physical and chemical properties and microbial communities; Table S4: Tolerance gene grouping information and KEGG orthology entries corresponding to each functional gene.
