# Supplementary material for: Microbial Assembly and Stress-Tolerance Mechanisms in Salt-Adapted Plants Along the Shore of a Salt Lake: Implications for Saline–Alkaline Soil Remediation
Source: Microorganisms. 2025 Aug 20;13(8):1942. doi: 10.3390/microorganisms13081942 (PMC12388437; doi:10.3390/microorganisms13081942)
Supplement: Supplementary file 1 [file microorganisms-13-01942-s001.zip › microorganisms-3777780-supplementary.pdf]

Supplementary Materials for

Microbial Assembly and Stress-Tolerance Mechanisms in Salt-Adapted Plants Along the Shore of a Salt Lake: Implications for Saline–Alkaline Soil Remediation

Xiaodong Wang [1], Liu Xu [1], Xinyu Qi [2], Jianrong Huang [3,*], Mingxian Han [1], Chuanxu Wang [4,5], Xin Li [6] and Hongchen Jiang [1,*]

1 State Key Laboratory of Geomicrobiology and Environmental Changes, China University of Geosciences, Wuhan 430074, China; shaodong@cug.edu.cn (X.W.); xuliuu@cug.edu.cn (L.X.); hanmingxian@cug.edu.cn (M.H.)
2 School of Environmental Science, China University of Geosciences, Wuhan 430074, China; 20231002846@cug.edu.cn
3 School of Life Sciences, Henan University, Kaifeng 475001, China
4 College of Life Sciences, Yuncheng University, Yuncheng 044000, China; wangchuanxu@ycu.edu.cn
5 Shanxi Key Laboratory of Yuncheng Salt Lake Ecological Protection and Resource Utilization, Yuncheng University, Yuncheng 044000, China
6 Department of Biology, Xinzhou Normal University, Xinzhou 034000, China; lixin1981@xznu.edu.cn
* Correspondence: huangjianrong@henu.edu.cn (J.H.); jiangh@cug.edu.cn (H.J.)

Contents of this file

Number of pages: 11

Number of figures: 6

Supplementary Figures

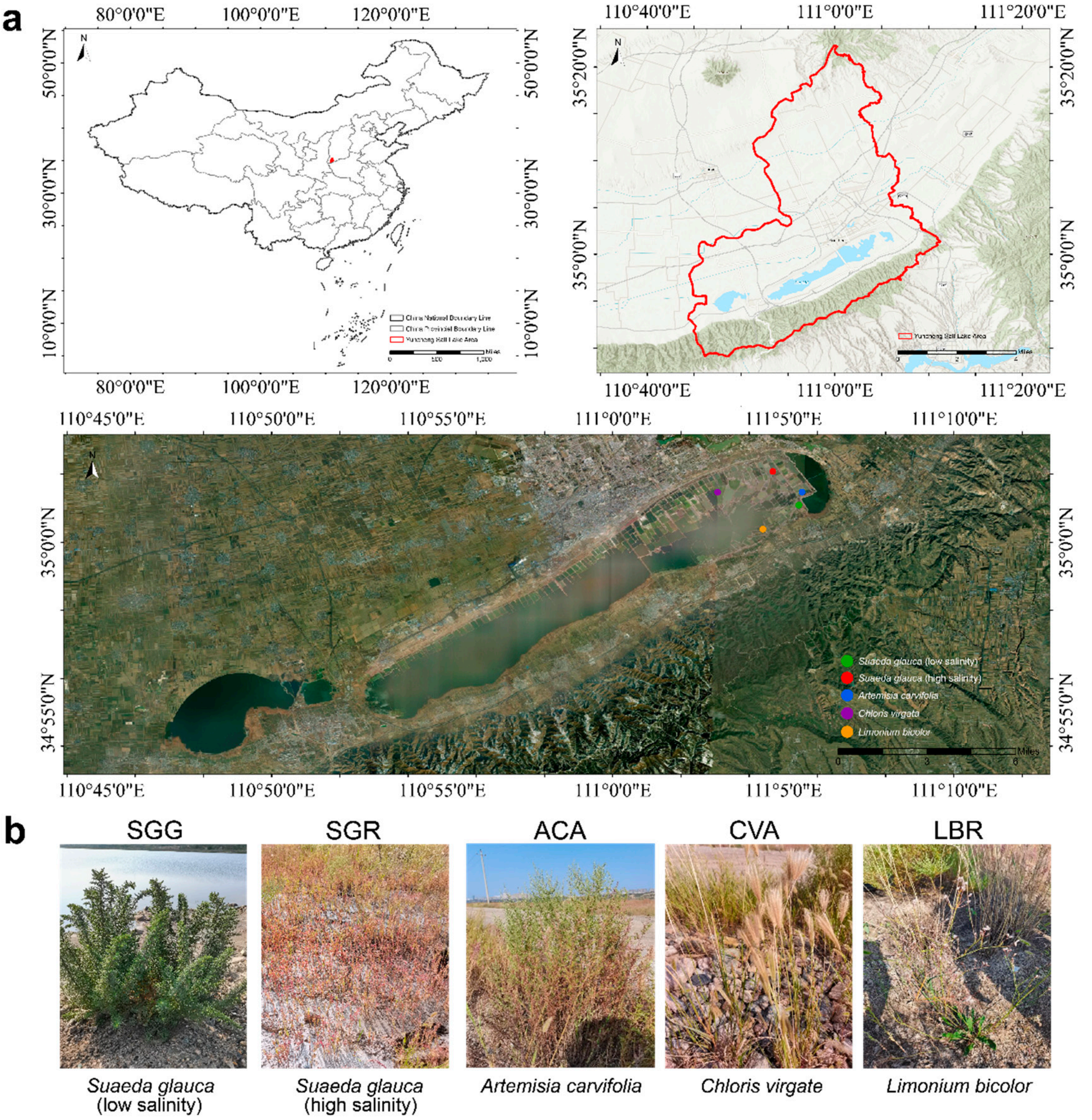

Figure S1. Information about collected samples. (a) Spatial distribution of the sampling sites. The location of each rhizosphere soil sample is denoted by a distinct color-coded marker. (b) Photographic representations of the host plants associated with the five collected rhizosphere soil samples in the Yuncheng Salt Lake region. Abbreviations above each image correspond to the respective plant rhizosphere soil samples.

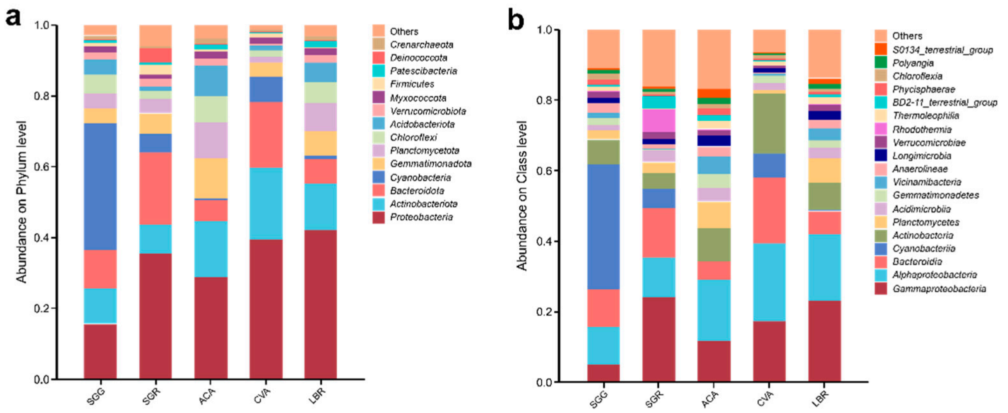

Figure S2. Relative abundances of the major bacteria phyla (a) and class (b) identified in the rhizosphere soils of five plant species.

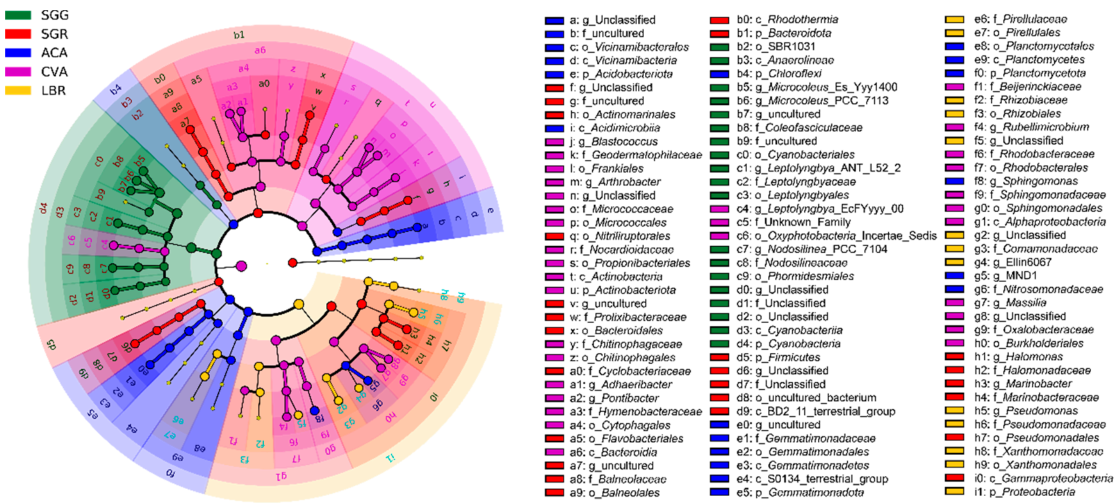

Figure S3. LEfSe analysis of the rhizosphere soils among five plants. Different colored regions represented different constituents. The inner to outer circle corresponds to the level of the phylum to the genus. (Significant differences were determined at LDA > 4).

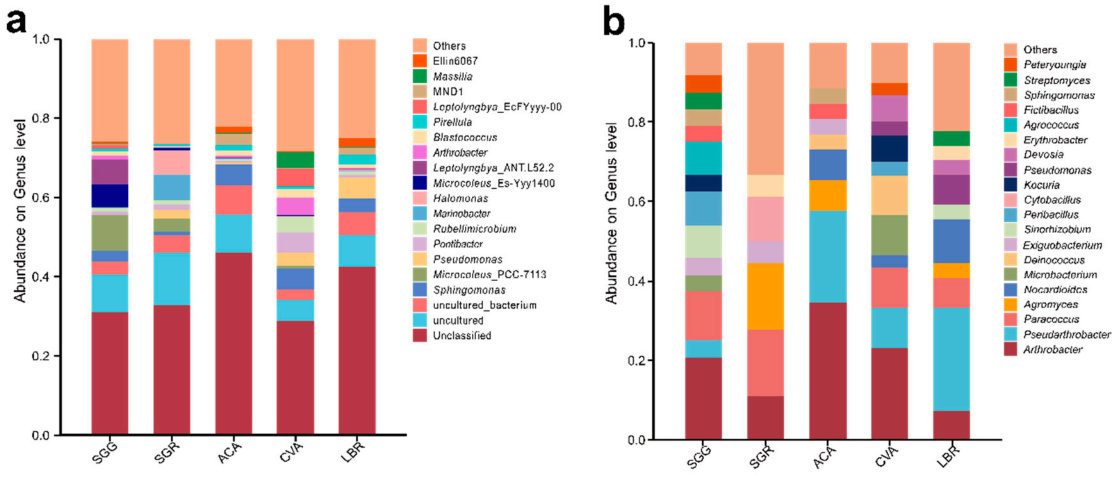

Figure S4. Taxonomic composition of rhizosphere microbial communities at the genus level. (a) Relative abundance of bacterial genera in rhizosphere soils associated with five plant species. (b) Relative abundance of culturable microbial isolates obtained from five rhizosphere soil samples.

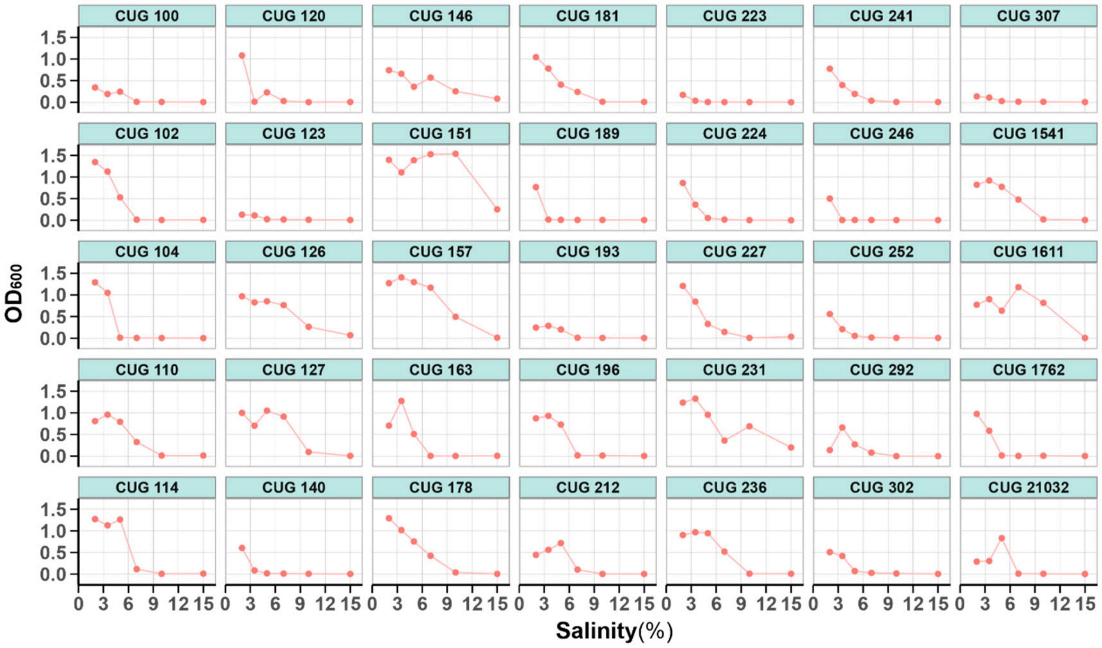

Figure S5. The OD_{600} values of 35 representative strains were measured after seven days of cultivation under five salinity gradients (2%, 3.5%, 5%, 7%, 10%, and 15%).

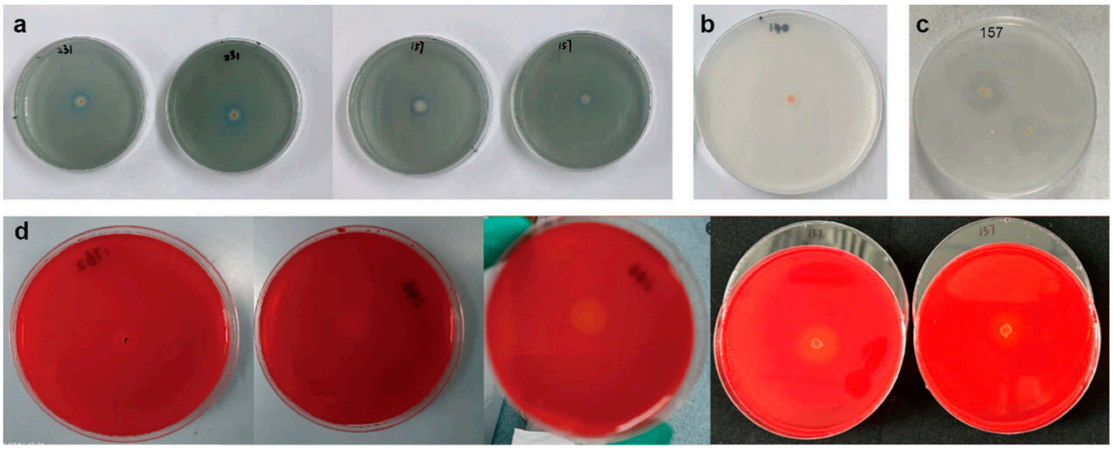

Figure S6. Representative plate photographs of bacterial strains exhibiting (a) siderophore production, (b) nitrogen fixation, (c) phosphate solubilization, and (d) cellulase synthesis capabilities.

Table S1. Formulations of nine culture media for microbial enrichment in rhizosphere soil.

Number	Recipe (/L)
1	NaCl, 0.2 g; Glucose, 0.5 g; Tryptone, 0.01 g; 10 mL of ($Na_2SO_4 \bullet 10H_2O$ 0.02 g; $MgSO_4 \bullet 7H_2O$, 0.02g; KBr 0.02 g, K_2HPO_4 0.01 g; KH_2PO_4, 0.01 g; $CaCl_2$, 0.02 g; $NaHCO_3$, 0.02 g; KNO_3, 0.01 g; H_2O, 100 mL);
2	NaCl, 0.2 g; Yeast extract, 0.5 g; Tryptone, 0.01 g; 10 mL of ($Na_2SO_4 \bullet 10H_2O$, 0.02 g; $MgSO_4 \bullet 7H_2O$, 0.02 g; KBr, 0.02g, K_2HPO_4, 0.01 g; KH_2PO_4, 0.01 g; $CaCl_2$, 0.02 g; $NaHCO_3$, 0.02 g; KNO_3, 0.01 g; H_2O, 100 mL);
3	$Na_2SO_4 \bullet 10H_2O$, 0.01 g; K_2HPO_4, 0.01 g; $CaCO_3$, 0.002 g; KCl, 0.02 g, $FeSO_4$, 0.002 g; NaCl, 0. 2 g; KNO_3, 0.02 g; $MgSO_4 \bullet 7H_2O$, 0.005 g; NaF, 0.001 g; KBr, 0.1 g; H_3BO_3, 0.002 g; peptone, 0.1 g;
4	$NaHCO_3$, 0.005 g; $MgCl_2$, 0.05 g; $CaCl_2$, 0.01 g; $ZnSO_4 \bullet 7H_2O$, 0.001 g; $FeCl_3 \bullet 6H_2O$, 0.003 g; $MnCl_2$, 0.001 g; $CaCl_2$, 0.002 g; $Na_2SO_4 \bullet 10H_2O$, 0.01 g; KBr 0.1 g; $MgCl_2$, 0.005 g; Yeast extract, 1 g; Casein acids hydrolysate, 0.75 g;
5	KNO_3, 2 g; $MgSO_4 \bullet 7H_2O$, 0.05 g; K_2HPO_4, 2 g; $CaCl_2$, 1 g; $FeSO_4$, 10 mg; Glucose, 10 g; Casein acids hydrolysate, 0.3 g;
6	NaCl, 2g; KCl, 3 g; K_2HPO_4, 1 g; KNO_3, 1 g; $MgCl_2$, 5 g; $MnCl_2 \bullet 4H_2O$, 0.02 g; $ZnSO_4$, 0.07 g; $FeSO_4 \bullet 7H_2O$, 0.02 g; Glycerol, 5 g; Fucose, 5 g; Asparagine, 0.5 g; Vitamin B1, 0.2 mg; Inositol, 0.5 mg; Vitamin C, 0.2 mg;
7	1/10 of ASW medium (Na_2SO_4, 4 g, KCl, 0.68 g; KBr, 0.1 g; H_3BO_3, 0.025 g; $MgCl_2$, 5.4 g; $CaCl_2 \bullet 2H_2O$, 1.5 g; $SrCl_2 \bullet 6H_2O$, 0.024 g; $NaHCO_3$, 0.2 g; Na_2HPO_4, 0.04 g; NH_4Cl_2, 0.5 g; NaF, 0.002 g, peptone, 5.0 g; Yeast extract, 1.0 g; pH 8.0). Water (/L): NaCl, 28.13 g; KCl, 0.77 g; $CaCl_2 \bullet 2H_2O$, 1.60 g; $MgCl_2 \bullet 6H_2O$, 4.80 g; $NaHCO_3$, 0.11 g; $MgSO_4 \bullet 7H_2O$, 3.50 g
8	Yeast Extract, 0.25 g; Peptone, 0.25 g; Casein hydrolysate, 0.25 g; Glucose, 0.25 g; Soluble Starch, 0.25 g; Sodium Pyruvate, 0.15 g; KH_2PO_4,0.15 g; $MgSO_4$, 0.012 g;
9	Tryptone, 5 g; Yeast extract, 2.5 g; NaCl, 5 g;

Table S2. Variation partitioning was used to assess the relative contribution of soil salinity, pH, and moisture to microbial community variation. The values of RDA1 and RDA2 correspond to the cosine of the angle between the environmental variable arrows and the ordination axes. r^2 measures the extent to which each environmental factor contributes to the variation in species distribution, while *P* (*p*-value) denotes the significance level of the correlation.

Parameter	RDA1	RDA2	R^2	*P*
pH	0.970	0.245	0.226	0.058
Salinity	0.997	-0.082	0.165	0.358
TOC	-0.115	-0.993	0.176	0.032
TP	0.164	-0.986	0.170	0.08
TN	-0.420	-0.908	0.161	0.295

Table S3. The Spearman correlation value between soil physical and chemical properties and microbial communities.

Property	pH	Salinity	TOC	TP	TN
Bacteroidia	0.00	0.35	0.83	0.47	0.33
Cyanobacteriia	-0.48	0.16	0.71	0.08	0.18
Chloroflexia	-0.44	-0.61	0.15	0.10	0.44
Alphaproteobacteria	-0.03	-0.15	0.07	0.49	0.70
Actinobacteria	-0.16	-0.55	0.18	0.46	0.62
Gammaproteobacteria	0.46	0.79	-0.12	0.29	-0.05
Acidimicrobiia	0.66	0.03	-0.68	-0.16	-0.43
Rhodothermia	0.72	0.41	-0.52	-0.30	-0.68
BD2-11_terrestrial_group	0.73	0.29	-0.55	-0.44	-0.78
Gemmatimonadetes	-0.15	-0.85	-0.29	-0.15	0.17
Vicinamibacteria	-0.26	-0.66	-0.69	-0.28	0.01
Thermoleophilia	-0.19	-0.63	-0.62	-0.19	0.10
Planctomycetes	0.11	-0.19	-0.85	-0.43	-0.39
Longimicrobia	0.15	-0.37	-0.77	-0.19	0.01
Verrucomicrobiae	0.02	0.14	-0.33	-0.51	-0.51
Phycisphaerae	-0.17	-0.54	-0.43	-0.64	-0.44
Anaerolineae	-0.37	-0.35	-0.57	-0.54	-0.18
Polyangia	0.00	-0.39	-0.64	-0.51	-0.12

Table S4. Tolerance gene grouping information and KEGG orthology entries corresponding to each functional gene

Classification	KEGG Orthology entry
Heat shock gene	*HtpX* (K03799), *hslR* (K04762), *hspR* (hspR)
UV-resistant gene	*UvrA* (K03701), *uvrB* (K03702), *uvrC* (K03703), *uvrD* (K03657), *ruvB* (K03551), *ruvA* (K03550), *ruvC* (K01159)
Oxidative stress gene	*KatE* (K03781), manganese catalase (K07217), *cpo* (K00433), *soxR* (K13639), *SOD2* (K04564)
Osmotic gene	*otsA* (K00697), *otsB* (K01087), *codA* (K17755), *betA* (K00108), *treY* (K06044), *treZ* (K01236), *opuAA* (K05847), *ABCB-BAC* (K06147), *opuC* (K05845), *gabT* (K07250)
